# Fimasartan ameliorates renal ischemia reperfusion injury via modulation of oxidative stress, inflammatory and apoptotic cascades in a rat model

**DOI:** 10.25122/jml-2021-0154

**Published:** 2022-02

**Authors:** Weaam Abbas, Murooj Altemimi, Heider Qassam, Ahmed Abdul Hameed, Qassim Zigam, Lamaan Abbas, Majid Jabir, Najah Hadi

**Affiliations:** 1. Department of Pharmacology & Therapeutics, Faculty of Medicine, University of Kufa, Kufa, Iraq; 2. Department of Pharmacology & Therapeutics, Faculty of Medicine, Jabir Ibn Hayyan Medical University, Najaf, Iraq; 3. Department of Pharmacology, Al-Mustaqbal University College, Babylon, Hilla, Iraq; 4. Al-Sadr Medical City, Al-Najaf Health Directorate, Al-Najaf Al-Ashraf, Iraq; 5. Department of Applied Science, University of Technology, Baghdad, Iraq

**Keywords:** Fimasartan, renal ischemia, Bcl-2, caspase 3, oxidative stress, IRI – ischemia-reperfusion injury, IP – intraperitoneal, TAC – total antioxidant capacity, ROS – reactive oxygen species, AKI – acute kidney injury, ARB – angiotensin II receptor blocker

## Abstract

Ischemia-reperfusion injury (IRI) can be defined as changes in the functions and structures of the tissues resulting from the restoration of blood after a period of ischemia. This study aimed to assess the potential protective effect of Fimasartan (angiotensin receptor antagonist) in the bilateral renal IRI in male rats through its potential effect on renal functions, modulation of the inflammatory cascade, oxidative stress, and apoptotic effect. The animals were equally assigned into four groups. The sham (negative control) group was exposed to surgical conditions without induction of IRI. The control group was exposed to ischemia by occluding the renal pedicles by clamps for 30 min, followed by restoration of blood for 2h. The vehicle-treated group received dimethyl sulfoxide (DMSO) by intraperitoneal injection (IP) 30 minutes before clamping. Fimasartan-treated group: rats pretreated with Fimasartan a dose of 3 mg/kg IP; this was half hour before occluding the renal pedicles. Animals were then exposed to 30 min ischemia (clamping the renal pedicles) followed by 2h reperfusion by releasing the clamps. Blood samples were collected to examine the levels of serum urea and creatinine. Renal tissue was used to measure the levels of cytokines (TNFα, IL-6) and total antioxidant capacity (TAC). Immunohistochemistry was used to assess the levels of Bax, caspase 3, and Bcl-2. Histopathological analyses were performed to detect the parenchymal injury. The present study shows that pretreatment with Fimasartan improves kidney function through its effects on oxidative stress, cytokines, and apoptotic markers.

## Introduction

Ischemia-reperfusion injury (IRI) is a pathological condition in the ischemic tissues that suffer from a low blood supply followed by reestablishment of blood flow resulting in further organ damage [1]. Blood flow restoration following ischemia causes a serious cell injury, including production of the reactive oxygen species (ROS), inflammatory responses, oxidative stress, and apoptosis [2, 3]. IRI deteriorates many organs, particularly the kidney, resulting in increased mortality rate injury. IRI is considered a major contributor to chronic renal failure and end-stage renal failure. Different conditions in which the kidneys are exposed to IRI include vascular and cardiac surgery, chronic renal artery stenosis, embolism trauma, atherosclerosis, and kidney transplantation [4–6]. More than 60% of patients with acute kidney injury (AKI) are due to IRI or acute tubular necrosis [7]. Annually, more than 1.5 million individuals die because of AKI, thereby discovering new therapeutic agents with low adverse effects can save the life of these patients [8]. Furthermore, AKI causes a long stay in hospitals, resulting in pressure on the health care providers and an economic burden on health services [9]. Multiple molecular mechanisms underpin the renal IRI, including the inflammatory responses initiated by various inflammatory molecules such as cytokines, chemokines, and increased expression of adhesion molecules leading to tissue necrosis [10]. Immune cells play a critical role in the inflammation response by secreting the TNF-α responsible for regulating a variety of immune, inflammatory and hematopoietic responses [11]. In AKI, the inflammatory response is critical, resulting in an induction of IL-6; therefore, IL-6 can be a useful target in AKI through modulating its effect [12–15]. ROS is another factor playing a critical role in deteriorating the case scenario of IRI as they are released in both phases resulting in deterioration of the affected tissues. Accumulation of ROS causes a state of imbalance, leading to tissue hypoxia and intracellular acidosis due to the generation of lactate.

Furthermore, excessive increase of ROS is also because of the decreased activity of a variety of antioxidant molecules such as superoxide dismutase, catalase, and glutathione peroxidase. These events influence the mitochondrial respiratory chain activity and burst ROS, resulting in oxidative damage to the bimolecular, including proteins, lipids, and DNA, ending in apoptosis and cell death [16]. Apoptosis occurs in response to hypoxic stress, resulting from ischemia and ROS production from reperfusion [17]. This stress can initiate both apoptosis pathways, including the intrinsic and extrinsic mechanisms [6]. Bax and Bcl2 are apoptotic regulators that are key players of IRI apoptosis. Bcl2 has an anti-apoptotic effect resulting in inhibition of the cell apoptosis via antagonizing the mitochondrial membrane permeability. The apoptotic impact of Bax releases a variety of cytokines, thereby inducing cell apoptosis [18, 19]. Caspase 3 is an executioner caspase-activated by initiator caspase to cleave a wide spectrum of cellular proteins [20]. Fimasartan is an angiotensin II receptor blocker (ARB) representing the latest drug from the ARB group [21, 22]. It is licensed in some countries, for example, in Korea by the Korea Food and Drug Administration, to treat hypertension [21].

Fimasartan was developed by Boryung Pharmaceutical, a Korean company, and received approval in many other countries such as China, Singapore, Russia, and India. It has a safety profile, efficacy, and tolerability; it has good properties compared to other ARBs, in addition to pleiotropic effects [22]. In general, ARBs represent the highly used antihypertensive drugs and the first recommended class according to American and European guidelines [23]. In recent studies, Fimasartan has been found to provide therapeutic action in patients suffering from acute coronary syndrome by decreasing inflammation of the carotid atherosclerotic plaque [24]. Particularly the Renin angiotensin aldosterone system (RAAS) modulates the inflammatory process and plays a significant role in causing renal IRI by different effecting mechanisms [25–26]. Especially, Ang II mediates the oxidative condition, inflammatory cascade, and apoptosis of renal tissue [27–29].

## Materials and Methods

### Animal maintenance, preparation, treatment, and sacrifice

Animals were fed in a standard laboratory in the animal house of the Faculty of Science, University of Kufa. All experiments were performed in the laboratory of the Department of Pharmacology and Therapeutics and Middle Euphrates Unit for Cancer Research, Faculty of Medicine, University of Kufa, Najaf, Iraq. All procedures were reviewed by the Institutional Animal Care and Use Committee (IACUC), University of Kufa, Najaf, Iraq.

### Experimental design

Wistar albino rats were between eight and twelve weeks and weighted 220–260 gm. Animals were assigned to four groups (5 animals in each group). Sham (negative control group) was subjected to the same operation without ischemia and reperfusion. The control group was subjected to ischemia for 30 min and 2h reperfusion [30–31]. The vehicle-treated group was pretreated with dimethyl sulfoxide (DMSO) 30 min prior to the induction of ischemia and reperfusion [31]. Fimasartan group was treated with Fimasartan (3 mg/kg) 30 min before the induction of ischemia and reperfusion [32].

### Induction of renal IRI

Animals were anesthetized by intraperitoneal injection with ketamine (100 mg/kg) and xylazine (10 mg/kg). To ensure that animals were anesthetized, reflex monitoring, including tail and leg pinching, was performed [33]. A midline incision was performed, and two renal pedicles were occluded for 30 min using clamps. At the time of ischemia, animals were maintained at 37°C using a heating pad. After 30 min of ischemia, the clamps were released, allowing the blood to resort for 2h and closing the abdomen. Normal saline (1 ml) was immediately injected into the animals to maintain fluid balance [34]. When the experiment ended, animals were sacrificed, and the left kidney was removed. The renal tissues were excised into two parts; the frozen one was used for the tissue assessment of cytokines and antioxidant readouts. The second part was put in 10% formalin for histopathological and immunohistochemistry analysis.

### Fimasartan preparation

Fimasartan was from Med Chem Express, USA Company. Molecular Formula: C_27_H_31_N_7_OS, Chemical Names: Fimasartan (BR-A-657), CAS.NO 247257-48-3. To prepare the drug, the powder was dissolved in DMSO and immediately used.

### Assessment of the renal function

The blood was collected from the heart and put in test tubes for 30 min to clot at room temperature. The blood samples were centrifuged at 3000 rpm for 15 min. The supernatant was collected and used to assess the levels of urea and creatinine using commercial kits.

### IL-6, TNF-α and total antioxidant capacity (TAC) measurement in kidney using ELISA

To measure the cytokines and antioxidant markers in the renal tissues, the frozen part of the kidney was washed with cold PBS to remove the blood. The renal tissue was weighted, and PBS containing 1% Triton X-100 and 1% protease cocktail inhibitor was added to the tissue in a ratio of 1:9 W/V, using an appropriate test tube. The samples were homogenized using an ultrasonic liquid processor [35]. The samples were spun down at 3000 rpm for 20 min at 4°C. The supernatant was used to measure the levels of TNFα, IL-6, and TAC using ELISA (Bioassay Technology Laboratory, China).

### Tissue preparation for histopathology

The renal tissue in the formalin was washed with cold normal saline to remove the clots. The tissue was processed in paraffin blocks. The tissue sections included the renal cortex and pelvis. Slices of 5 μM thickness were cut and stained with hematoxylin and eosin stain. The changes in the renal tissues were investigated and included the following: cellular swelling, eosinophilic cast, tubular dilation, development of proteinaceous cast, desquamation of epithelial cells, inflammatory cells infiltration, and necrosis [36]. The renal tissue was investigated using a microscope with magnification lenses from 100 to 400X. The investigation was performed by an independent pathologist unaware of the experimental groups. Histopathological changes were scored from 0 to 4 according to the percentages of affected tubules as follow: the normal tissue was assigned 0, score 1: less than 25%, score 2: 25–50%, score 3: 50–75% and score 4: 75–100% [37].

### Immunohistochemistry (IHC) study

To measure the levels of BAX, Bcl-2, and caspase 3 in the renal tissue, immunohistochemistry analysis was used. The immunostaining was performed in the paraffin-embedded tissue sections (5 μM thickness). Briefly, the sections were deparaffinized, and the endogenous peroxidase activity was blocked using 3% (v/v). Non-specific binding sites can be reduced by incubating the tissue sections in serum-free proteins. The tissue sections were incubated overnight at 4°C with primary antibodies purchased from the Bioassay Technology Laboratory against BAX (1:100), Bcl-2 (1:100), or caspase 3 (1:100). The sections were then washed for 1h and incubated with a biotinylated secondary antibody for 30 min at 37°C. The tissue sections were then washed and incubated with horseradish peroxidase for 30 min followed by incubation with chromogen for 15 min (100 μL per slide). The tissue sections were then counterstained with hematoxylin [38]. The immunostaining of the BAX, Bcl-2, and caspase 3 was quantified using a Q-score system in which the scores were calculated by multiplying the immunostaining intensity and positive stain area. The labeling intensity was graded as follows: score 0: no staining, score 1: weak staining, score 2: moderate staining, and score 3: strong staining. The stained cells were represented as percentages ranging from 0–100% [39]. An independent pathologist unaware of the study design investigated the immunostained tissue sections.

### Statistical analysis

SPSS software version 26.0 was used to analyze the data. The results were represented as mean±SEM unless otherwise stated. Analysis of variance (ANOVA) was used to compare the groups, followed by a post hoc test [40]. Finally, the Kruskal-Wallis test was used to assess the mean differences among the study groups in terms of morphological changes and immunohistochemistry. In this study, the P≤0.05 is considered statistically significant.

## Results

### Influence of Fimasartan on renal function

**Figure 1.**
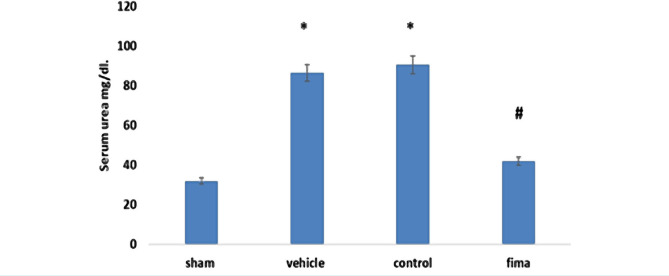
Mean level of serum urea among the groups. Data are represented as mean±SEM, n=5. Statistical analysis was performed using a one-way ANOVA followed by a post hoc test. * – P<0.05 compared to the sham group; #– P<0.05 compared to control and vehicle-treated groups.

**Figure 2.**
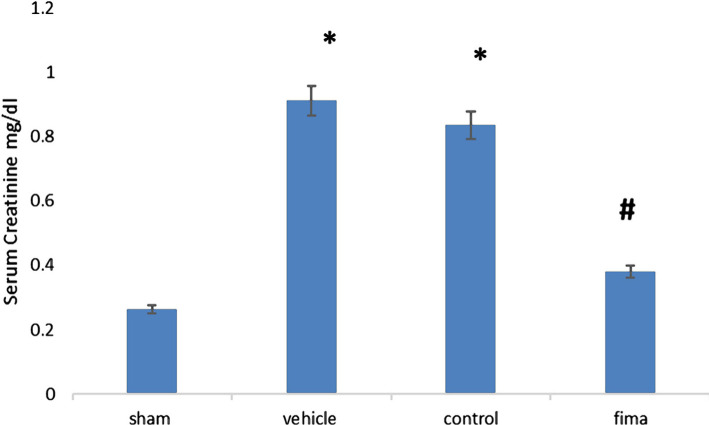
Mean level of serum creatinine among the groups. Data are expressed as mean±SEM, n=5. Statistical analysis was performed using a one-way ANOVA followed by a post hoc test. * – P<0.05 compared to the sham group; #– P<0.05 compared to control and vehicle-treated groups.

The results showed that serum urea and creatinine levels were higher in control and vehicle-treated groups than in sham groups. In contrast, pretreatment with Fimasartan resulted in a marked decrease in serum urea and creatinine levels compared to the control and vehicle-treated, Figures 1 and 2.

### Influence of Fimasartan on oxidative stress in the kidney (TAC)

**Figure 3.**
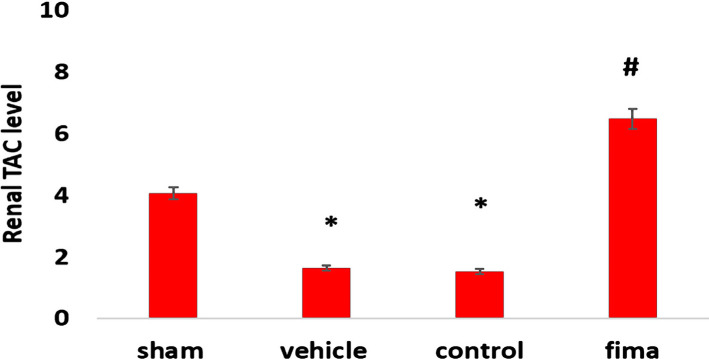
Tissue levels of TACin the kidney (U/ml) among the group. Data are represented as mean±SEM, n=5. Statistical analysis was performed using a one-way ANOVA followed by a post hoc test. * – P<0.05 compared to the sham group, # – P<0.05 compared to control and vehicle-treated groups.

The data in control and vehicle-treated groups showed a dramatic decrease in the levels of renal (TAC) compared to the sham group (Figure 3). On the other hand, pretreatment with Fimasartan resulted in a marked elevation in TAC levels compared to the control and vehicle-treated groups (Figure 3).

### Influence of Fimasartan on renal cytokines (TNFα, IL-6)

**Figure 4.**
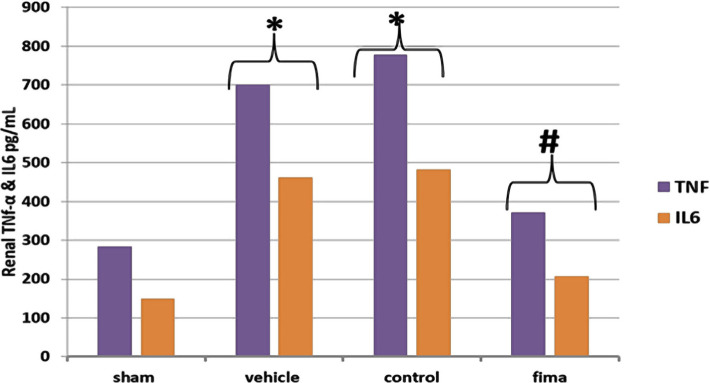
Mean level of serum creatinine among the groups. Data are expressed as mean±SEM, n=5. Statistical analysis was performed using a one-way ANOVA followed by a post hoc test. * – P<0.05 compared to the sham group; #– P<0.05 compared to control and vehicle-treated groups.

The levels of TNFα and IL-6 in the kidney for the control and vehicle-treated groups increased significantly compared to the sham group. By contrast, these levels significantly dropped in the Fimasartan-treated group versus the control and vehicle-treated groups (Figure 4).

### Histopathological examination

**Figure 5.**
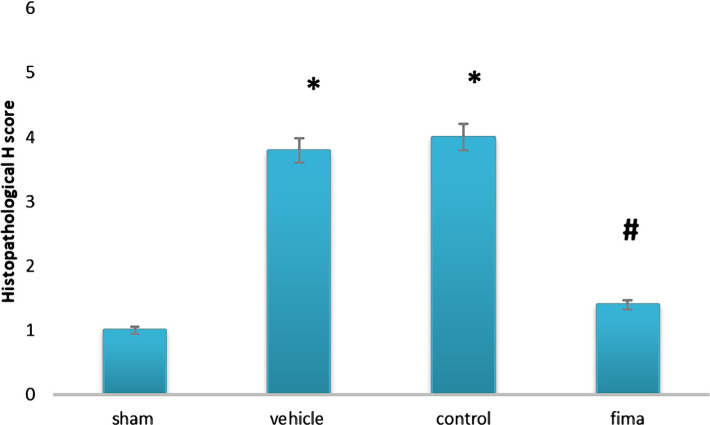
Histopathology scores of the study groups. Statistical analysis was performed using a Kruskal-Wallis test, n=5. * – P<0.05 compared to the sham group; # – P<0.05 compared to control and vehicle-treated groups.

**Figure 6.**
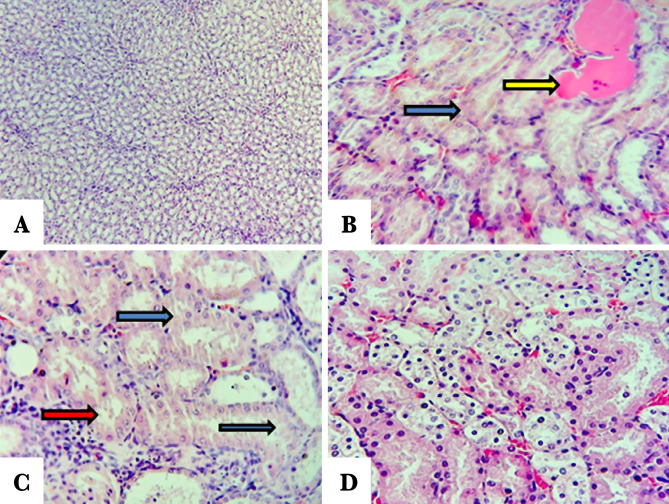
Representative pictures of the renal tissue sections stained with hematoxylin and eosin. A – Sham negative group reveals normal morphology; B – Control group reveals cellular swelling, increased cytoplasmic eosinophilic (blue arrow), cast formation (yellow arrow); C – Vehicle-treated group shows a cellular injury including swelling, cytoplasmic eosinophilic (blue arrows), and interstitial inflammation (red arrow); D – Fimasartan-treated group showing a slight change in most areas of renal tissue.

The score of damage and morphological changes of kidneys among the groups were shown in Figures 5 and 6. The renal morphology was normal in the sham negative group (Figure 6A). In contrast, the histopathological investigation in the control and vehicle-treated groups showed tubular cell swelling, tubular dilation, and damage and degeneration of tubular structure. Furthermore, there is a cast formation and congestion of the lumen (Figure 6 B and C). Pretreatment with Fimasartan maintained the normal morphology of renal tissue. The treatment effect is characterized by a slight swelling of the renal tubules with mild interstitial congestion (Figure 6D).

### Immunohistochemistry finding

#### Influence of Fimasartan on the anti-apoptotic Bcl-2, proapoptotic Bax, and caspase 3

**Figure 7.**
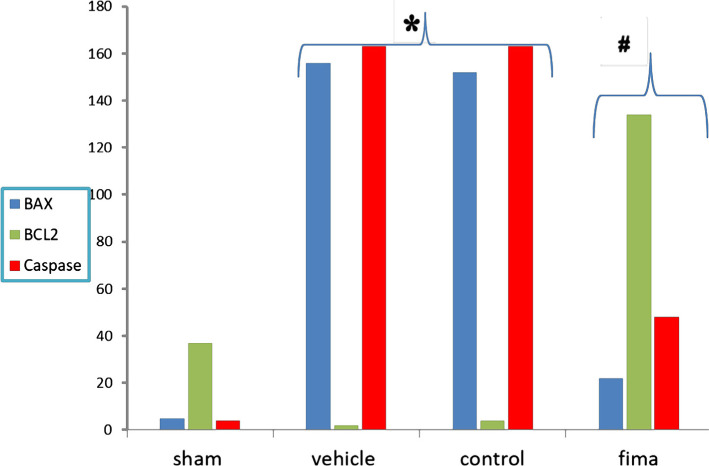
Q score of BAX, BCL-2, and Caspase 3 staining of the renal tissue among the four groups (N=5). Data are represented as mean±SEM, n=5. * – P<0.05 compared to the sham group; # – P<0.05 compared to control and vehicle-treated groups.

To investigate Bcl-2 expression, Bax, and caspase, immunohistochemistry was used. Q score was used to calculate the intensity of labeling of these molecules. The results showed that pretreatment with Fimasartan caused a marked increase in Q score of Bcl2 and decrease in Q score of Bax and caspase 3, compared to the control and vehicle-treated groups (Figure 7).

#### Influence of Fimasartanon the anti-apoptotic Bcl-2

**Figure 8.**
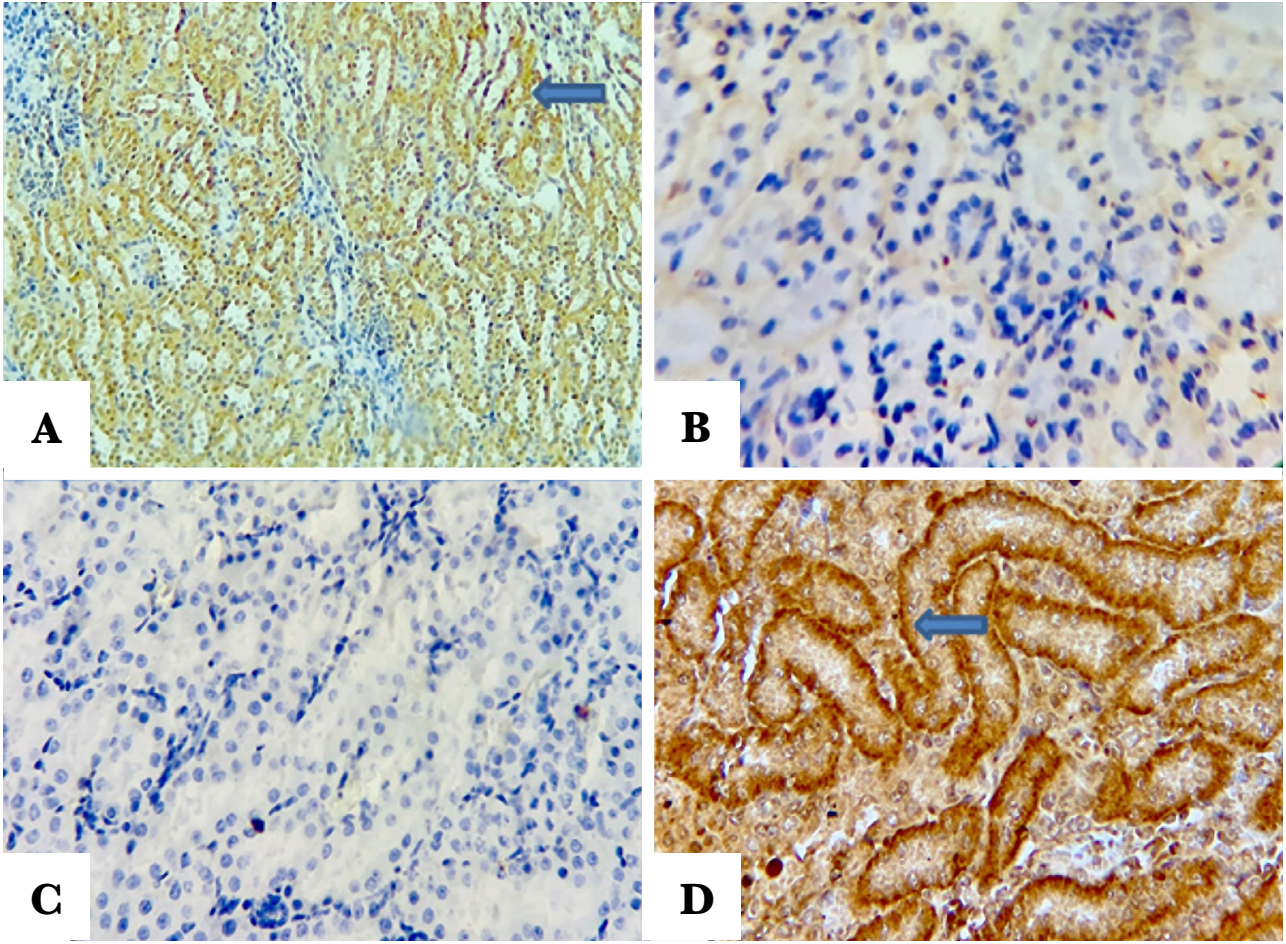
Representative pictures of renal tissue sections. A – Sham negative group show positive BCL-2 labeling intensity (indicated by arrow); B – Control group showing a negative stain; C – Vehicle-treated group shows negative stain; D – Fimasartan-treated group reveals positive labeling intensity BCL-2 (indicated by arrow).

Renal tissue was probed with anti Bcl2 antibody to investigate the Bcl2 expression. Pretreatment with Fimasartan revealed strong labeling intensity (brown color) compared to the control and vehicle-treated groups (Figure 8 A, B, C, and D).

#### Influence of Fimasartan on Bax

**Figure 9.**
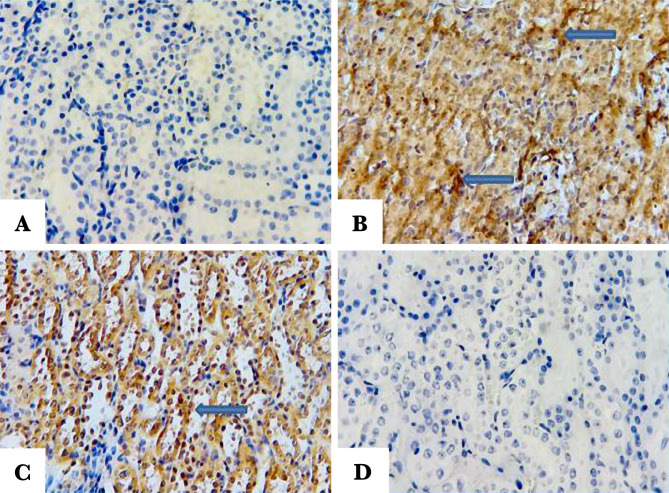
Representative pictures showing the renal tissue sections of the study groups. A – Sham negative group shows negative Bax labeling intensity; B – Control group reveals positive labeling intensity (indicated by arrow); C – Vehicle-treated group shows positive labeling intensity (indicated by arrow); D – Fimasartan-treated group showing negative Bax stain.

To investigate the expression level of Bax, the renal tissue was probed with anti-Bax antibody. The tissue sections of the sham negative group and Fimasartan-treated group revealed no staining of the Bax, (Figure 9 A and D). By contrast, the tissue sections of the control and vehicle-treated groups were stained positively (darkly brown color), Figure 9 B and C.

#### Influence of Fimasartan on the proapoptotic caspase3

**Figure 10.**
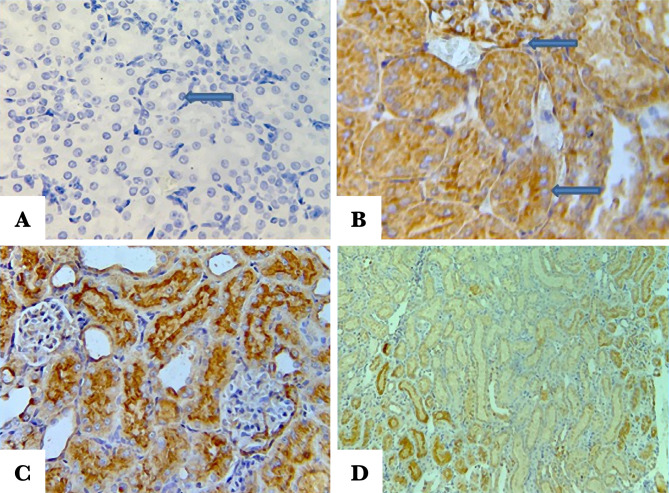
Representative pictures of the renal tissue sections. A – Sham negative group reveals negative caspase 3 labeling intensity; B – Control group reveals positive caspase 3 labeling intensity (brown color); C – Vehicle-treated group shows positive caspase 3 labeling intensity; D – Fimasartan-treatment group showing negative caspase 3 labeling intensity.

To investigate the expression level of caspase 3, the renal tissue was probed with an anti-caspase 3 antibody. The tissue sections of the sham negative group and Fimasartan-treatment group revealed no staining of caspase 3 (Figure 10 A and D). On the other hand, the control and vehicle-treated groups were positively stained (brown stain), Figure 10 B and C.

## Discussion

The present study demonstrated significantly high urea and creatinine levels in the control and vehicle-treated groups compared to the sham group. The observed increase in these markers could be attributed to renal tissue damage and decreased glomerular capacity [33]. Pretreatment with Fimasartan reduced the urea and creatinine Fimasartan levels compared to the control and vehicle-treated groups highlighting the potential protective effect of Fimasartan. This finding was also reported by Cho *et al.* (2018). This result may be explained by the fact that Fimasartan antagonizes AT1 receptors, thereby reducing the deterioration of the renal tissue [40, 41]. The histopathological findings of the current study revealed a significantly lower degree of tissue injury in the Fimasartan-treated group compared to control and vehicle-treated groups. This finding was reported by a previous study [41]. This low degree of injury by Fimasartan treatment is likely to be related to its ability to decrease oxidative stress and inhibit most pro-inflammatory responses [42–45], resulting in a decrease in the movement of the inflammatory cell to ischemic tissue [46]. Prior studies have noted the importance of TNF-α and IL-6 in the molecular pathogenesis of IRI. The results of this study indicate that the TNF-α and IL-6 levels were significantly lower in the Fimasartan-treatment group than in the control and vehicle-treated group. These results agree with those obtained by Cho *et al.* (2018) [41]. A possible explanation for this might be that Fimasartan could act as an IKK inhibitor, thereby inhibiting the NF-κB. This effect could reduce the accumulation and recruitment of the inflammatory cells in the ischemic tissues [47]. These results reflect those of Lee *et al.* [48], who found that Fimasartan decreases macrophage number, decreases plague breaking, and enhances plaque stabilization. Shigeoka *et al.* showed that NLRP3 could signal the injury responses in the renal epithelium [49]. It can thus be suggested that pretreatment with Fimasartan inhibits NLRP3 inflammasome and protects the kidney through modulation of the immune response and cytokine response. The present study demonstrated a significantly high level of TAC in the Fimasartan-treated group in comparison to control and vehicle groups. This result agrees with Kim *et al.* that revealed that Fimasartan significantly increased the antioxidant enzyme in unilateral ureteral obstruction in mice by up regulating expression of the mRNA of NQO1 and HO-1, the protein expression of those genes, as well as of CuSOD, MnSOD, and catalase [50].

Furthermore, Nezu and Suzuki [51] established the activation of anti-oxidative transcription factor Nrf2 in the renal tubule, diminishing the reactive oxygen species protecting them from damage and fibrosis, this mechanism being involved via Fimasartan action [52]. The current study showed a marked decrease in apoptotic regulators (Bax and caspase 3) and an increase in levels of Bcl2 in the Fimasartan-treated group compared to the control and vehicle groups. These results are in line with previous studies [53] that established ARBs lead to upregulation of Bcl2 levels and decreasing Bax and caspase 3 expression. These outcomes can be explained via the impact of Fimasartan, which decreases apoptosis cells by blocking the AT1 receptor, inhibiting proapoptotic (p53-Bax), and increasing anti-apoptotic (Bcl2) [54].

## Conclusion

This study showed that Fimasartan reduces the levels of the inflammatory molecules TNF-α and IL-6, increases the expression levels of anti-apoptotic Bcl2, and decreases the expression levels of proapoptotic Bax and caspase 3. Furthermore, this study also showed that Fimasartan ameliorates the histopathological changes that occur in response to renal IRI. Taken together, these results suggest that Fimasartan could be a protective agent in renal IRI through its effect on inflammation, oxidative stress, and apoptosis regulators.

## Acknowledgements

### Conflict of interest

The authors declare no conflict of interest.

### Ethical approval

The study was reviewed and approved by the Institutional Animal Care and Use Committee (IACUC), University of Kufa, Najaf, Iraq (ID: EC230).

### Authorship

WA, MA, AAH, LA contributed to the methodology and writing the original draft. NH contributed to conceptualizing, study design, and editing the manuscript. HQ contributed to editing the manuscript,and QZ, MJ contributed to data curation and analysis.
